# Longitudinal follow-up of health effects among workers handling engineered nanomaterials: a panel study

**DOI:** 10.1186/s12940-019-0542-y

**Published:** 2019-12-09

**Authors:** Wei-Te Wu, Lih-Ann Li, Tsui-Chun Tsou, Shu-Li Wang, Hui-Ling Lee, Tung-Sheng Shih, Saou-Hsing Liou

**Affiliations:** 1 National Institute of Environmental Health Sciences, National Health Research Institutes, Miaoli County, Taiwan; 2 Institute of Environmental and Occupational Health Sciences, National Yang Ming University, Taipei, Taiwan; 3 Department of Chemistry, Fu Jen Catholic University, Taipei, Taiwan; 4 Institute of Labor, Occupational Safety, and Health, Ministry of Labor, Taipei, Taiwan; 5 Division of occupational medicine, Division of fanily medicine, Department of Family and Community Medicine, Tri-Service General Hospital, National Defense Medical Center, Taipei, Taiwan

**Keywords:** Nanomaterials, health effect markers, follow-up study, control banding, panel study

## Abstract

**Background:**

Although no human illness to date is confirmed to be attributed to engineered nanoparticles, occupational epidemiological studies are needed to verify the health effects of nanoparticles. This study used a repeated measures design to explore the potential adverse health effects of workers handling nanomaterials.

**Methods:**

Study population was 206 nanomaterial-handling workers and 108 unexposed controls, who were recruited from 14 nanotechnology plants. They were followed up no less than two times in four years. A questionnaire was used to collect potential confounders and detailed work conditions. Control banding was adopted to categorize risk level for each participant as a surrogate marker of exposure. Health hazard markers include cardiopulmonary dysfunction markers, inflammation and oxidative damage markers, antioxidant enzymes activity, and genotoxicity markers. The Generalized Estimating Equation model was applied to analyze repeated measurements.

**Results:**

In comparison to the controls, a significant dose-dependent increase on risk levels for the change of superoxide dismutase (*p*<0.01) and a significant increase of glutathione peroxidase change in risk level 1 was found for nanomaterial-handling workers. However, the change of cardiovascular dysfunction, lung damages, inflammation, oxidative damages, neurobehavioral and genotoxic markers were not found to be significantly associated with nanomaterials handling in this panel study.

**Conclusions:**

This repeated measurement study suggests that there was no evidence of potential adverse health effects under the existing workplace exposure levels among nanomaterials handling workers, except for the increase of antioxidant enzymes.

## Background

Nanotechnology is regarded as one of the most important industries currently, and the use of nanomaterials in industrial and consumer applications continues to grow. Engineered nanoparticles are now being used in many different types of commercially available products ranging from electronics, to medical and health care products, food, textiles, athletic gear, and household products [1, 2]. The use of nanomaterials in the development of novel materials for new purposes has led to a rapid increase in the numbers of workers exposed to engineered nanoparticles [1, 2]. Although nanotechnology has been applied to many different domains, the potential risks generated from its application are often neglected [2, 3].

There is a need to assess the risk of potentially adverse health effects among workers handling nanomaterials. Most of the documents about the toxicities of engineered nanoparticles have come mainly from animal or in vitro studies. The health effects induced by engineered nanoparticles in animal inhalation studies include pulmonary fibrosis, granuloma, and inflammation, cardiovascular effects, oxidative stress, and pleural plaque formation, lung cancer, and mesothelioma-like effects [2–8]. In addition, needle-like fibrous carbon nanotubes induces an asbestos-like granuloma formation and increases the likelihood of mesothelioma in a tumor prone mouse strain [9]. A recent multi-day, full-shift sampling study among 108 U.S. workers presents unrelated evidence between different metrics of carbon nanotubes and nanofibers and clinically relevant outcomes, included lung function, resting blood pressure, resting heart rate, and complete blood count components or pulmonary symptoms [10]. However, another study in the same research team shows that inhalable Carbon nanotubes and nanofibers structures were associated with matrix metalloproteinase-2 (MMP-2), interleukin-18, glutathione peroxidase (GPx), myeloperoxidase, and superoxide dismutase (SOD) in sputum, and MMP-2, matrix metalloproteinase-9, metalloproteinase inhibitor 1/tissue inhibitor of metalloproteinases 1, 8-hydroxy-2′-deoxyguanosine, GPx, SOD, endothelin-1, fibrinogen, intercellular adhesion molecule 1, vascular cell adhesion protein 1, and von Willebrand factor in blood [11]. It implies that the selection of health effect markers (acute or chronic) induced by engineered nanoparticles and using which specimens to test is important.

Most of the evidence for the health effects of engineered nanoparticles on humans was generated from unintentionally produced ultrafine particles. Evidence of human health effects of ultrafine particles (lung inflammation, oxidative damage, worsening of heart disease, atherosclerosis, asthma, and possibly lung cancer) came from air pollution epidemiological studies of unintentionally produced ultrafine particles that were generated from traffic pollution and combustion products, such as diesel exhaust and welding fumes [5, 6]. Epidemiological studies have shown a positive correlation between the particulate matter concentrations in air pollution and increased morbidity and mortality in adults and children [5, 6]. In addition to healthy individuals, populations having impaired health conditions may become more susceptible to developing health problems from ultrafine particles exposure [5, 6].

Although health effects caused by engineered nanoparticles have never been confirmed in humans, there is accumulating evidence from animal studies supporting that exposure to some nanomaterials is harmful. Assessment of risks of engineered nanoparticles exposure requires information on critical target organs and knowledge of toxic endpoints gathered from studies in which relevant exposure routes and exposure levels have been used [12, 13]. Workers involved in the handling of engineered nanomaterials are likely exposed to engineered nanoparticles through inhalation. However, published information on exposures in the workplace is sparse. The main reason for this sparseness is that measuring exposure to engineered nanoparticles is not an easy task [1, 2, 13–17]. The behaviors and characteristics of EN differ in several ways from traditional aerosols [2, 3]. There is still insufficient scientific evidence in regards to the effect of engineered nanoparticles has on health. There is not enough information to determine the range of particle sizes, exposure parameters of the engineered nanoparticles (that should be measured in order to characterize exposure), and which instruments and methods are the most appropriate to use [13–17].

Control banding method has become an acceptable alternative to face with uncertainties relating to nanomaterial exposure [12, 18]. Hazard (severity) bands are generated based on toxicologic data of nanomaterials combined with exposure (probability) bands that reflect the exposure levels. Control banding is a semi-quantitative assessment of risk that offers minimal preventive measures to be implemented according to the estimated levels of risk.

There is no consensus on methodologies for exposure assessment because occupational epidemiological research on engineered nanoparticles is largely lacking. Although we have reported that some adverse health effects may be attributed to engineered nanoparticles exposure [19, 20], longitudinal epidemiological studies still need to be conducted in order to verify the toxic endpoints among workers exposed to engineered nanoparticles. The objective of this exploratory study was to further explore the health effects of workers who handle engineered nanoparticles.

## Materials and Methods

### Study design

This exploratory study applied a longitudinal design with repeated measurements. This panel study design had five repeated examinations completed within an interval of four years. Nanomaterial-handling workers were recruited from 14 nanomaterials manufacturing and/or using plants in Taiwan. The non-exposed controls were selected from workers who did not handle nanomaterials and who worked at the same plants. This was done in order to have comparable geographic areas and socioeconomic statuses. Blood, urine, and exhaled breath condensate specimens were repeatedly collected at baseline, 6 months later, 18 months later, 30 months later, and 42 months later to measure selected biomarkers after informed consent was obtained from individual participant. Physical examination of pulmonary function, heart rate variability, neurobehavioral test and self-administered questionnaire were also repeatedly performed. This study has been approved by the institutional review board of the National Health Research Institutes of Taiwan.

### Study population

We have conducted an industrial hygiene survey of the nanotechnology plants in Taiwan [21]. We estimated that about 70 plants were manufacturing and/or using nanomaterials in Taiwan. Among them, 39 plants were visited and invited to participate in this epidemiological study. Fourteen plants agreed to participate in this study. Some physicochemical properties and work conditions of nanomaterials collected from 14 plants except for one research institute are listed in Table 1.

**Table 1:** Some physicochemical properties and work conditions of nanomaterials handled in these 13 factories

Factory number	Type of nanomaterial handling	Nanomaterial handled	Major nanomaterial handled	Size (nm)	Amount handled (mg/time)	Duration of handling (hour/time)	Frequency of handling (times/week)	Type of nanomaterials handled	Total population	Control group	Exposed group
1	Use	Nano-silver	Nano-silver	Unknown	20	5	5	LS	9	0	9
2	Use	Fe_2_O_3_	Fe_2_O_3_	6-10	5000	2	1	LS	4	0	4
		Nano-gold		3-40	19	0.1	1	LS			
		Nano-silver		5-10	2.1	0.1	1	LS			
	Mfg	Fe_2_O_3_	Fe_2_O_3_	6-10	5000	0.2	8	LS			
		Nano-gold		3-40	10	0.25	8	LS			
		Nano-silver		5-10	21	0.1	8	LS			
3	Use	Titanium dioxide	Titanium dioxide	15-20	10000	2.5	2.5	Powder and LS	35	10	25
	Mfg	Titanium dioxide	Titanium dioxide	15-20	1000000	1	8	Powder and LS			
4	Use	Nano-silver	Nano-silver	Commercial secret	Commercial secret	3	7	LS	2	1	1
	Mfg	Nano-silver	Nano-silver	Commercial secret	Commercial secret	7	3	LS			
5	Use	Titanium dioxide	Titanium dioxide	20	50000	4	1	LS	7	3	4
6	Use	Silicon dioxide	Silicon dioxide	10	50000	1	1	LS	14	5	9
		Nano-silver		100	50	1	1	LS			
7	Use	Carbon nanotube	Carbon nanotube	40	100	1	1	Powder and LS	10	1	9
		Silicon dioxide	Silicon dioxide	100	50000	1	1	Powder			
8	Use	Carbon nanotube	Carbon nanotube	0.5	Commercial secret	Commercial secret	Commercial secret	LS	29	10	19
9	Use	Silicon dioxide	Silicon dioxide	12-17	300	0.2	4	Powder	27	11	16
10	Mfg	Silicon dioxide	Silicon dioxide	160	2500	5	1	LS	26	11	15
		Silicon dioxide		100-200	60000	5	8	Colloid			
11	Use	Carbon nanotube	Carbon nanotube	110	5000	0.5	4.5	Powder, LS, and gel	43	25	18
	Mfg	Carbon nanotube	Carbon nanotube	110	4000000	4	12	Powder and LS			
12	Use	Carbon nanotube	Carbon nanotube	100	>20	1	3.5	Powder	34	7	27
		Nano-silver		>100	>40	1	3.5	LS			
13	Use	Titanium dioxide	Titanium dioxide	Unknown	5	6	0.33	LS	14	4	10

Abbreviation: *mfg* Manufacturing, *LS* Liquid suspension

We performed five repeat examinations during the four years. A total of 496 workers, including 258 exposed workers and 238 controls, were recruited in this study cohort. Those who have participated in no less than two examinations were included in the data analysis. 314 workers were examined no less than two times, including 206 exposed workers and 108 controls. The response rate of total workers was estimated to be 314/496=63.3%. The response rate for exposed group was 206/258=79.8%, while it was 108/238=45.3% for control group.

### Exposure assessment

Since there is still a lack of consensus on equipment and methodology for personnel sampling of engineered nanoparticles, this study used the control banding nanotool risk level matrix that was proposed by Dr. Paik and his colleagues [12, 18] to categorize the risk level of each participant as a surrogate marker of exposure. The risk level matrix was calculated based on the probability scores of the exposure and the severity scores of the nanomaterial toxicity. The variables considered in the exposure probability were collected from individual questionnaire, including the estimated amount of material used (25 points), dustiness/mistiness (30 points), number of employees with similar exposure (15 points), frequency of operation (15 points), and duration of operation (15 points). The factors considered in the calculation of the severity score include nanomaterial (70% of severity score) and parent material (30% of severity score). The factors considered in the calculation of the severity score of the nanomaterials were collected from industrial survey of individual factory, including surface chemistry (10 points), particle shape (10 points), particle diameter (10 points), solubility (10 points), carcinogenicity (6 points), reproductive toxicity (6 points), mutagenicity (6 points), dermal toxicity (6 points), and asthmagenicity (6 points). The factors considered in the calculation of the severity score of parent material include occupational exposure limit (10 points), carcinogenicity (4 points), reproductive toxicity (4 points), mutagenicity (4 points), dermal toxicity (4 points), and asthmagenicity (4 points).

In order to obtain consistent scores, the nanomaterial toxicity severity score was based on the toxicity summary tables of a review document [22]. The factors of exposure probability score was based on the questionnaires collected from individual worker exposed to the various nanomaterials. The cross-table of the severity scores (band) and probability scores (band) was used to generate the risk levels (1 to 4) for each individual. The higher the risk levels, the higher the risk of health effects.

### Health effect markers

Based on the review of the inhalation studies in humans and animals [2, 4–8, 23–28], this study investigated six aspects of potential toxic endpoints, including lung inflammation, oxidative damage or lipid peroxidation and antioxidant enzyme activity, cardiovascular diseases markers, DNA damage and genotoxicity, pulmonary function, and neurobehavioral function. Each marker was measured according to standard protocols that were either provided by suppliers or developed by laboratories.

The markers measured for each aspect of health effects include:

1. Inflammation markers, such as Clara cell protein (CC16) [29], heat shock protein 70 [30], nitric oxide (NO) [31, 32], nuclear factor κB (NFkB) transcription factor activation [33].
2. Oxidative damage markers and antioxidant enzyme activities: such as copper-zinc superoxide dismutase (SOD), glutathione peroxidase-1 (GPX-1) [34, 35], 8-hydroxydeoxyguanosine (8-OHdG) [36, 37], N^7^-methyl guanosine (N7-MedG) [37], and isoprostane (8-iso-prostaglandin F2α) (PGF2α) [38].
3. Cardiovascular markers, such as fibrinogen, vascular cell adhesion molecule (VCAM), intercellular adhesion molecule-1 (ICAM-1), interleukin-6 (IL-6), IL-6 soluble receptor (IL6sR) [30, 39], myeloperoxidase (MPO), arylesterase, paraoxonase (PON 1) [40], high-sensitivity C-reactive protein (hsCRP) [41], and heart rate variability (HRV) (including time domain such as standard deviation of all normal to normal R-R intervals (SDNN), root mean square of successive differences between adjacent normal cycles (RMSSD) and frequency domains such as very low frequency (VLF), low frequency (LF), high frequency (HF), ratio of LF to HF (LF/HF)) [42].
4. Genotoxicities using the comet assay, including %DNA in the tail, tail moment, olive moment, and L/H ratio (tail to head ratio) [43], and micronucleus (MN) assay [44].
5. Lung function, including forced vital capacity (FVC), forced expiratory volume at 1 second (FEV1.0), maximal mid-expiratory flow (MMF), peak expiratory flow rate (PEFR), forced expiratory flow at 25% (FEF25%), forced expiratory flow at 50% (FEF50%), and forced expiratory flow at 75% (FEF75%) [45].
6. Neurobehavioral tests, including reaction time test and 5, 6, 7-digit forward and backward memory tests [46].

### Data analysis

We used SPSS statistical software version 20 (SPSS, Chicago, IL, USA). Means and standard deviations were used to describe the distributions of continuous variables. Percentages were used to describe the distributions of categorical variables. The chi-square test was used to test the differences among the categorical variables. *P* < 0.05 was considered statistically significant.

The generalized estimating equation (GEE) model for repeated measurements was used in this data analysis. We have performed five repeated examinations during the four-year. Those who participated in no less than two examinations were included in this data analysis. Two types of analyses were performed. First, the difference in the change of markers among five repeated measurements was analyzed based on the risk level (Risk Level 1*Time vs. control*Time, and Risk Level 2*Time vs. control*Time). Second, the dose-response relationship of change of markers on continues risk levels (Risk Level*Time) was analyzed by trend test. To control for confounding variables, this study first identified potential confounders for each health effect marker. Those variables associated with health effect markers were identified and considered as confounders and adjusted in the GEE model. Since smoking is a strong confounder, it was forced to be adjusted for every effect marker.

In addition to confounders, the study also collected data on potential background exposure to incidental ultrafine particles. It was found that there was no difference among risk level 2, risk level 1, and the control groups regarding frequencies of ultrafine particles exposure resulting from smoking, second-hand smoke, transportation, residential exposure close to traffic roads, residential exposure close to industrial factories (within 50 meters), burning incense in the house, and burning anti-mosquito coils in the house. Therefore, this study did not adjust for incidental ultrafine particles exposure in the final GEE model.

## Results

### Physicochemical properties of nanomaterials handled in these 13 factories

The participating factories include photocatalyst manufacturing, nanomaterials manufacturing, toilet manufacturing, air cleaner manufacturing, light emission device manufacturing, nanopaint manufacturing, colorants manufacturing, carbon nanotube manufacturing, textile manufacturing, and self-cleaning tiles manufacturing factories. These factories were created to handle nanomaterials as early as 2001. The size of nanotechnology factories used in the study was small and the number of employees ranged from 1 to 27. Most factories used local ventilation as a method to control dust emission. Most used water flushing and sweeping to clean the leakage of nanomaterials.

Table 1 shows the physicochemical properties and work condition of nanomaterials in these 13 plants. One factory manufactured nanomaterials only, eight factories used nanomaterials only, and four factories both manufactured and used nanomaterials. The types of nanomaterials handled in these plants include nanosilver, silicon dioxide, titanium dioxide, carbon nanotube, and other nanomaterials. The major types of nanomaterials handled in these factories were silicon dioxide, titanium dioxide, and CNT. The size of nanomaterials handled was usually less than 100 nm.

The amount of nanomaterials handled varied. Some factories handled only a few milligrams, some handled hundreds of grams, and some even handled kilograms. The duration of handling nanomaterials ranged from 0.1 to 7 hours per time. Most of the duration of handling time was less than 1 hour per time. The frequency of handling was usually once per week. Some of factories handled nanomaterials more often than once per week (Table 1).

The physical state of nanomaterials handled was frequently liquid suspension, and powder combined with liquid solutions. Only three factories handled nanomaterial powder. The powder handled included two silicon dioxide powders and one carbon nanotube powder (Table 1).

### Distribution of characteristics among study population

The researchers performed five repeated examinations during four-year follow-up. 134 individuals participated in the first examination, which include 99 exposed workers (53 in risk level 1 (RL1), 43 in risk level 2 (RL2), and 3 in risk level 3 (RL3)) and 35 controls. 225 individuals participated in the second examination, including 153 exposed workers (84 RL1, 64 RL2, and 4 RL3) and 72 controls. 220 individuals participated in the third examination, including 150 exposed workers (76 RL1, 68 RL2, and 6 RL3) and 70 controls. 213 individuals participated in the fourth examination, including 126 exposed workers (61 RL1, 60 RL2, and 5 RL3) and 87 controls. 175 individuals participated in the fifth examination, including 94 exposed workers (48 RL1, 40 RL2, and 6 RL3) and 81 controls. Those who participated in no less than 2 examinations were included in the data analysis. Therefore, the total study population was 314 workers, which included 206 exposed workers and 108 controls who were examined no less than twice.

There were 108 RL1, 93 RL2 and 5 RL3 in the exposed group. This study combined RL3 with RL2 as RL2 in the data analysis. The characteristics of the study participants stratified by risk level are shown in Table 2. Education, alcohol drinking and betel nut chewing differed significantly by risk levels. Risk level 2 had a higher educational level, more alcohol drinkers and more betel nut chewers, while the control group had more university educated subjects, less alcohol drinkers, and less betel nut chewers. The difference in age, gender, ethnic distribution, and smoking status among the three groups was not significant. The mean length of employment was 8 to 11 years. The mean duration of follow-up was about 2.05 years.

**Table 2:** Demographic distribution in study population

Variables	Control (*n*=108)		RL1 (*n*=108)		RL2 & RL3 (*n*=98)		
	*n*	%	*n*	%	*n*	%	*p-*value^a^
Age							
≦36yrs	53	49.07	62	57.41	57	58.16	0.34
>36yrs	55	50.93	46	42.59	41	41.84	
Gender							
Female	29	26.85	23	21.30	18	18.37	0.33
Male	79	73.15	85	78.70	80	81.63	
Ethnicity							
Taiwanese or South Fujienese	85	78.70	89	82.41	73	74.49	0.36
Hakkanese	16	14.81	9	8.33	17	17.35	
Others	7	6.48	10	9.26	8	8.16	
Education							
Senior high school	19	17.59	14	12.96	20	20.41	<0.01
College	62	57.41	47	43.52	33	33.67	
Institute or higher education	27	25.00	47	43.52	45	45.92	
Smoking							
No	95	87.96	96	88.89	77	78.57	0.07
Yes	13	12.04	12	11.11	21	21.43	
Alcohol Drinking							
No	101	93.52	103	95.37	81	82.65	<0.01
Yes	7	6.48	5	4.63	17	17.35	
Chewing betel nut							
No	107	99.07	108	100.00	93	94.90	0.02
Yes	1	0.93	0	0.00	5	5.10	

^a^Chi-square analysis

### Nanomaterials handled

The types of nanomaterials handled by the 206 exposed individuals were silicon dioxide, titanium dioxide, carbon nanotubes, nanosilver, and other nanomaterials including nanoresins, nanogold, nanoclay, nanoalumina, and metal oxides. Most frequent type of exposure was mixed types of nanomaterials (*n* = 121, 58.7%). Silicon dioxide was the most frequent single exposure (*n* = 88, 42.7%), followed by titanium dioxide (*n* = 70, 34.0%), carbon nanotubes (*n* =68, 33.0%), nanosilver (*n* =38, 18.4), and others (*n* = 63, 30.6%).

### Health effects of nanomaterials handling workers

There were no significant differences between the exposed and the control workers in changes of lung inflammation markers such as serum Clara cell 16 (CC16), exhaled breath nitric oxide, serum nuclear factor κB transcription factor activation, and nuclear factor κB transcription factors activation in exhaled breath condensate (EBC) (Table 3). There were also no significant differences between exposed workers and controls in the changes of oxidative damage or lipid peroxidation markers, such as urine 8-hydroxydeoxyguanosine, urine N^7^-methyl guanosine, plasma 8-hydroxydeoxyguanosine, and isoprostane (8-iso-prostaglandin F2α) in exhaled breath condensate (Table 3).

**Table 3:** Generalized estimating equation analysis of ≥ 2 repeated measurements of inflammatory, oxidative damage and lipid peroxidation markers over 4 years

	Control		Risk Level 1		Risk Level 2		GEE analysis coefficient *B (SE)*		
							Model 1		Model 2
Variables^a.^	*N*	Mean ± SD	*N*	Mean ± SD	*N*	Mean ± SD	RL1*Time	RL2*Time	RL*Time
FeNO (ppb) 2nd	70	22.07 ± 12.94	81	18.93 ± 9.5	66	23.59 ± 14.79	0.16 (0.80)	-1.05 (0.96)	-0.55 (0.48)
FeNO (ppb) 3rd	72	19.81 ± 13.09	73	15.95 ± 8.14	69	20.33 ± 11.58			
FeNO (ppb) 4th	78	13.33 ± 8.31	67	12.32 ± 8.12	65	15.88 ± 11.02			
FeNO (ppb) 5th	65	16.99 ± 11.56	52	17.97 ± 10.3	55	17.1 ± 11.51			
CC16(ng/ml) 1st	34	5.03 ± 1.42	50	5.77 ± 1.85	44	5.17 ± 1.54	0.26 (0.157)	0.33 (0.149) ^★^	0.17 (0.074) ^★^
CC16 (ng/ml) 2nd	73	5.7 ± 2.41	81	5.27 ± 2.06	67	5.14 ± 2.19			
CC16 (ng/ml) 3rd	73	9.01 ± 2.76	74	9.52 ± 3.08	72	9.06 ± 3.52			
CC16 (ng/ml) 4th	79	6.29 ± 2.26	68	6.39 ± 2.77	65	6.02 ± 2.71			
CC16 (ng/ml) 5th	63	4.46 ± 2.61	51	4.02 ± 3.24	56	4.18 ± 2.68			
NF-kB (Serum) (pg/ml) 1st	33	0.53 ± 0.22	51	0.54 ± 0.2	43	0.6 ± 0.24	0.029 (0.026)	0.016(0.028)	0.001(0.014)
NF-kB (Serum) (pg/ml) 2nd	73	1.09 ± 0.64	82	0.85 ± 0.52	67	0.85 ± 0.58			
NF-kB (Serum) (pg/ml) 3rd	67	0.73 ± 0.22	71	0.79 ± 0.27	69	0.74 ± 0.24			
NF-kB (Serum) (pg/ml) 4th	77	0.48 ± 0.12	66	0.5 ± 0.16	65	0.48 ± 0.14			
NF-kB (Serum) (pg/ml) 5th	63	1.05 ± 0.4	52	1.12 ± 0.45	57	1.03 ± 0.42			
NF-kB (EBC) (pg/ml) 1st	33	0.9 ± 0.46	52	0.88 ± 0.46	46	0.8 ± 0.42	-0.045 (0.025)	-0.047 (0.024)	-0.024 (0.012) ^★^
NF-kB (EBC) (pg/ml) 2nd	75	0.71 ± 0.27	81	0.63 ± 0.2	68	0.64 ± 0.24			
NF-kB (EBC) (pg/ml) 3rd	73	0.87 ± 0.33	74	1.03 ± 0.35	71	1.02 ± 0.34			
NF-kB (EBC) (pg/ml) 4th	77	0.56 ± 0.17	68	0.54 ± 0.15	64	0.55 ± 0.11			
NF-kB (EBC) (pg/ml) 5th	65	1.19 ± 0.44	52	1.13 ± 0.52	57	1.08 ± 0.45			
Urine 8-OHdG (ng/mL) 1st	35	6.96 ± 4.09	53	7.9 ± 3.83	45	8.68 ± 4.09	-0.242 (0.200)	-0.092 (0.210)	-0.06 (0.104)
Urine 8-OHdG (ng/mL) 2nd	69	4.78 ± 3.07	77	4.43 ± 2.76	66	4.03 ± 3.07			
Urine 8-OHdG (ng/mL) 3rd	71	3.48 ± 2.55	74	3.05 ± 1.89	73	3.36 ± 2.73			
Urine 8-OHdG (ng/mL) 4th	78	4.96 ± 4.43	67	5.71 ± 4.3	63	6.83 ± 4.83			
Urine 8-OHdG (ng/mL) 5th	66	3.85 ± 2.68	50	4.46 ± 2.72	56	4.1 ± 3.04			
plasma 8-OHdG (pg/mL) 1st	19	18.63 ± 9.62	31	15.11 ± 8.83	29	18.38 ± 10.67	-0.162 (0.810)	-0.596(0.694)	-0.301(0.350)
plasma 8-OHdG (pg/mL) 2nd	73	17.26 ± 13.67	76	17.9 ± 11.99	65	20.75 ± 15.23			
plasma 8-OHdG (pg/mL) 3rd	73	16.42 ± 6.7	73	16.53 ± 6.8	70	16.96 ± 6.69			
plasma 8-OHdG (pg/mL) 4th	79	13.78 ± 6.04	67	14.95 ± 7.31	64	14.51 ± 6.35			
plasma 8-OHdG (pg/mL) 5th	64	16.12 ± 6.86	50	15.06 ± 5.84	57	18.77 ± 7.87			
EBC 8-isoPGF2 (pg/mL) 1st	34	5.56 ± 2.01	51	5.21 ± 1.65	46	5.25 ± 1.73	0.565 (0.180) ^★★^	0.341 (0.170) ^★^	0.179 (0.084) ^★^
EBC 8-isoPGF2 (pg/mL) 2nd	72	4.15 ± 2.02	82	3.96 ± 1.61	66	4.2 ± 1.65			
EBC 8-isoPGF2 (pg/mL) 3rd	72	11.36 ± 6.88	74	11.5 ± 7.55	72	12.62 ± 7.46			
EBC 8-isoPGF2 (pg/mL) 4th	78	4.38 ± 2.19	67	4.58 ± 2.61	65	4.5 ± 2.35			
EBC 8-isoPGF2 (pg/mL) 5th	65	2.45 ± 1.76	52	2.37 ± 1.67	57	2.52 ± 1.79			
Urine N7-MedG (ug/mL) 3rd	71	2.23 ± 1.98	74	2.1 ± 1.81	73	2.22 ± 1.99	0.430 (0.408)	0.362 (0.457)	0.198 (0.225)
Urine N7-MedG (ug/mL) 4th	78	5.72 ± 3.67	67	7.13 ± 4.9	64	6.43 ± 4.01			
Urine N7-MedG (ug/mL) 5th	66	5.81 ± 4.34	52	7.42 ± 4.58	56	5.41 ± 4.23			

^a.^times of measurements (1st: at baseline; 2nd: at 6 months later; 3rd: at 18 months later; 4th: at 30 months later; 5th: at 42 months later)

★★*p*<0.01; ★0.01<*p*<0.05

Analytical Model (Dependent variable: FeNO) includes main effects of sex, smoking, asthma, and rhinitis.

Analytical Model (Dependent variable: CC16) includes main effects of sex, smoking, and chewing betel nut.

Analytical Model (Dependent variable: NF-kB (Serum)) includes main effects of sex, smoking, and education.

Analytical Model (Dependent variable: NF-kB (EBC)) includes main effects of sex, and smoking.

Analytical Model (Dependent variable: Urine 8-OHdG) includes main effects of sex, smoking, valvular heart disease, and folliculitis.

Analytical Model (Dependent variable: plasma 8-OHdG) includes main effects of sex, and smoking.

Analytical Model (Dependent variable: EBC 8-isoPGF2) includes main effects of sex, and smoking.

Analytical Model (Dependent variable: Urine N7-MedG) includes main effects of sex, smoking, chewing betel nut, chronic bronchitis, and pigmentation.

GEE analysis showed that only the change of antioxidant enzyme activity in the exposed group was significantly different from control group (Table 4). The change of superoxide dismutase (SOD) over the five repeated measurements in RL2 of the exposed group were significantly greater than in controls (*p*<0.01, Table 4). In addition, the GEE analysis revealed a significant dose-dependent increase on risk levels for the change of SOD (*p*<0.01). The increase in the change of glutathione peroxidase-1 (GPx) over the five repeated measurements in RL1 of the exposed group were significantly greater than in controls (*p*<0.01, Table 4), but were not significant for RL2 and trend test.

**Table 4:** Generalized estimating equation analysis of ≥ 2 repeated measurements of antioxidant enzymes, DNA damage and reaction time over 4 years

	Control		Risk Level 1		Risk Level 2		GEE analysis coefficient B (SE)		
							Model 1		Model 2
Variables ^a.^	N	Mean ± SD	N	Mean ± SD	N	Mean ± SD	RL1*Time	RL2*Time	RL1*Time
SOD (U/ml) 1st	34	7.98±2.17	53	8.61±3.55	45	8.11±3.5	0.487 (0.278)	0.720 (0.278) ^★★^	0.355 (0.136) ^★★^
SOD (U/ml) 2nd	74	9.48±5.04	82	7.67±5.1	68	7.79±5.96			
SOD (U/ml) 3rd	73	16.86±7.4	74	19.57±8.77	72	20.3±9.04			
SOD (U/ml) 4th	78	12.67±3.63	67	12.1±2.76	65	11.87±2.9			
SOD (U/ml) 5th	64	13.38±4.68	52	11.88±4.31	57	13.43±4.98			
GPx (nmol/min/ml) 1st	35	117.07±20.67	53	120.44±16.18	44	128.02±29.2	3.652 (1.319) ^★★^	1.612 (1.470)	0.759 (0.724)
GPx (nmol/min/ml) 2nd	74	95.52±26.18	82	80.47±24.87	68	82.97±25.18			
GPx (nmol/min/ml) 3rd	73	157.11±24.04	74	153.8±21.9	72	151.17±29.02			
GPx (nmol/min/ml) 4th	79	100.09±14.67	68	99.32±15.35	65	104.41±14.38			
GPx (nmol/min/ml) 5th	64	129.49±19.49	52	124.34±16.85	57	130.91±16.4			
%DNA in Tail 1st	35	13.43±11.74	53	16.69±9.84	46	14.82±8.34	2.280 (0.792) ^★★^	2.240 (0.794) ^★★^	1.075 (0.379) ^★★^
%DNA in Tail 2nd	75	33.98±21.37	82	22.47±15.01	68	21.69±20.91			
%DNA in Tail 3rd	73	34.68±22.19	74	26.83±20.59	72	32.28±21.77			
%DNA in Tail 4th	79	18.37±8.36	68	17.69±6.53	65	16.4±7.91			
%DNA in Tail 5th	63	16.06±5.27	52	18.01±6.19	57	15.59±5.25			
Tail Moment 1st	35	17.3±23.2	53	15.16±14.95	46	15.99±16.21	16.184 (3.802) ^★★^	14.495 (3.970) ^★★^	7.052 (1.790) ^★★^
Tail Moment 2nd	65	112.23±106.89	78	48.75±45.96	61	37.22±68.26			
Tail Moment 3rd	73	145.68±129.55	74	106.66±122.55	72	143.41±135.74			
Tail Moment 4th	79	35.16±35.07	68	29.16±26.23	65	26.93±29.46			
Tail Moment 5th	63	28.67±18.75	51	30.09±18.03	57	25.11±19.39			
Olive Moment 1st	35	8.92±10.45	53	8.8±7.37	46	8.42±6.8	6.188 (1.698) ^★★^	6.302 (1.678) ^★★^	2.990 (0.768) ^★★^
Olive Moment 2nd	61	43.81±42.99	77	21.71±15.58	59	12.96±12.98			
Olive Moment 3rd	73	77.18±73.51	74	53.61±67.1	72	73.93±75.04			
Olive Moment 4th	79	16.92±14.56	68	14.57±11.31	65	13.17±12.17			
Olive Moment 5th	63	13.77±7.11	51	14.65±7.64	57	12.93±9.18			
L/H ratio 1st	35	0.99±0.92	53	0.96±0.61	46	0.99±0.73	0.006 (0.036)	0.025 (0.035)	0.012 (0.018)
L/H ratio 2nd	74	1.08±0.87	81	1.02±0.9	68	0.81±0.86			
L/H ratio 3rd	71	0.7±0.67	73	0.51±0.63	71	0.66±0.7			
L/H ratio 4th	77	0.23±0.12	68	0.22±0.1	65	0.21±0.13			
L/H ratio 5th	63	0.2±0.08	50	0.21±0.08	57	0.19±0.08			

^a.^times of measurements (1st: at baseline; 2nd: at 6 months later; 3rd: at 18 months later; 4th: at 30 months later; 5th: at 42 months later)

★★*p*-values interaction term <0.01 in the GEE model

Analytical Model (Dependent variable: SOD) includes main effects of age, sex, smoking, chewing betel nut, and education.

Analytical Model (Dependent variable: GPx) includes main effects of sex, smoking, drinking, hypertension, and atopic dermatitis.

Analytical Model (Dependent variable: %DNA in Tail) includes main effects of age, sex, smoking, and education.

Analytical Model (Dependent variable: Tail Moment) includes main effects of sex, and smoking.

Analytical Model (Dependent variable: Olive Moment) includes main effects of sex, and smoking.

Analytical Model (Dependent variable: L/H ratio) includes main effects of sex, smoking, and atopic dermatitis.

There were no significant differences between the exposed and control workers in the changes of genotoxicity markers such as the comet assays and for the change of neurobehavioral test such as reaction time (Table 4).

No significant differences between the exposed and control workers were found for the changes of cardiovascular dysfunction markers, including fibrinogen, VCAM, ICAM, IL- 6, IL- 6sR, paraoxonase, arylesterase, myeloperoxidase, high-sensitivity C-reactive protein, and heart rate variability parameters including SDNN, RMSSD, VLF, LF, HF, and LF/HF (Table 5, 6 and 7).

**Table 5:** Generalized estimating equation analysis of ≥ 2 repeated measurements of cardiovascular disease markers over 4 years

	Control		Risk Level 1		Risk Level 2		GEE analysis coefficient B (SE)		
							Model 1		Model 2
Variables^a^	*N*	Mean ± SD	*N*	Mean ± SD	*N*	Mean ± SD	RL1*Time	RL2*Time	RL1*Time
Fibrinogen (mg/dL) 1st	35	263.56 ± 45.17	53	274.17 ± 49.14	46	286.77 ± 67.83	-3.420 (3.136)	-6.490 (3.494)	-3.248 (1.746)
Fibrinogen (mg/dL) 2nd	75	249.49 ± 43.28	80	263.08 ± 56.28	67	261.08 ± 51.43			
Fibrinogen (mg/dL) 3rd	72	219.14 ± 42.73	74	217.83 ± 42.33	72	225.28 ± 43.47			
Fibrinogen (mg/dL) 4th	75	213.5 ± 54.15	67	225.52 ± 57.38	63	218.51 ± 62.15			
Fibrinogen (mg/dL) 5th	64	221.2 ± 54.41	52	225.57 ± 61.48	57	219.59 ± 66.26			
VCAM (ng/mL) 1st	34	612.9 ± 151.48	53	590.67 ± 178.76	46	642.79 ± 203.85	-24.322 (10.469) ^★^	-33.739 (10.115) ^★★^	-16.788(4.937)
VCAM (ng/mL) 2nd	75	462.74 ± 160.74	81	516.37 ± 166.86	68	514.06 ± 164.67			
VCAM (ng/mL) 3rd	71	429.03 ± 127.83	72	426.52 ± 139.26	71	393.77 ± 131.75			
VCAM (ng/mL) 4th	79	372.81 ± 101.62	68	359.93 ± 100.05	64	375.79 ± 109.01			
VCAM (ng/mL) 5th	64	565.41 ± 106.74	52	563.16 ± 103.29	56	569.2 ± 126.19			
ICAM (ng/mL) 1st	35	505.42 ± 137.68	53	463.57 ± 136.36	46	498.75 ± 150.27	2.674 (8.871)	-0.024 (8.996)	0.243 (4.505)
ICAM (ng/mL) 2nd	75	443.67 ± 202.98	81	525.75 ± 197.94	67	538.21 ± 218.68			
ICAM (ng/mL) 3rd	73	421.23 ± 124.21	73	449.52 ± 137.34	71	435.78 ± 138.17			
ICAM (ng/mL) 4th	79	331.37 ± 128.31	68	338.84 ± 119.72	65	321.29 ± 132.41			
ICAM (ng/mL) 5th	64	222.29 ± 64.61	52	196.7 ± 54.22	56	240.85 ± 83.54			
hsCRP (mg/L) 1st	33	0.61 ± 0.55	51	0.72 ± 0.6	39	0.81 ± 0.92	-0.071 (0.044)	-0.038 (0.055)	-0.022 (0.276)
hsCRP (mg/L) 2nd	75	0.7 ± 0.77	74	0.76 ± 0.88	64	0.64 ± 0.71			
hsCRP (mg/L) 3rd	67	0.81 ± 0.9	69	1 ± 1.05	69	1.04 ± 1.08			
hsCRP (mg/L) 4th	76	0.75 ± 0.77	60	0.73 ± 0.8	61	0.92 ± 0.83			
hsCRP (mg/L) 5th	63	0.94 ± 0.97	46	1.06 ± 1.23	54	0.93 ± 0.94			

^a^times of measurements (1st: at baseline; 2nd: at 6 months later; 3rd: at 18 months later; 4th: at 30 months later; 5th: at 42 months later)

★★*p*<0.01; ★0.01<*p*<0.05

Analytical Model (Dependent variable: Fibrinogen) includes main effects of age, sex, smoking, chewing betel nut, and education.

Analytical Model (Dependent variable: VCAM) includes main effects of sex, and smoking.

Analytical Model (Dependent variable: ICAM) includes main effects of sex, smoking, and asthma.

Analytical Model (Dependent variable: hsCRP) includes main effects of sex, smoking, drinking, hypertension, and hyperlipidemia.

**Table 6:** Generalized estimating equation analysis of ≥ 2 repeated measurements of cardiovascular disease markers over 4 years

	Control		Risk Level 1		Risk Level 2		GEE analysis coefficient B (SE)		
							Model 1		Model 2
Variables^a^	*N*	Mean ± SD	*N*	Mean ± SD	*N*	Mean ± SD	RL1*Time	RL2*Time	RL1*Time
IL-6 (pg/mL) 1st	35	9.9 ± 4.04	53	9.27 ± 3.34	45	9.81 ± 4.2	-0.314 (0.285) ^★★^	-0.759 (0.276)	-0.355 (0.136) ^★★^
IL-6 (pg/mL) 2nd	74	4.35 ± 3.29	82	5.45 ± 3.83	66	5.54 ± 3.95			
IL-6 (pg/mL) 3rd	73	2.79 ± 3.03	73	6.11 ± 11.35	72	3.95 ± 8.53			
IL-6 (pg/mL) 4th	79	3.89 ± 7.51	68	3.51 ± 7.77	65	1.71 ± 2.2			
IL-6 (pg/mL) 5th	46	3.06 ± 2.29	41	2.49 ± 2.4	44	2.04 ± 1.61			
IL-6 sR (ng/mL) 1st	35	37.88 ± 11.76	53	35.64 ± 10.1	46	34.79 ± 10.65	0.523 (0.752)	0.809 (0.700)	0.377 (0.348)
IL-6 sR (ng/mL) 2nd	75	46.45 ± 11.87	82	40.72 ± 11.04	68	41.14 ± 11.87			
IL-6 sR (ng/mL) 3rd	73	33.87 ± 13.35	73	32.02 ± 12	72	33.77 ± 12.01			
IL-6 sR (ng/mL) 4th	79	31.52 ± 9.91	68	31.63 ± 8.87	65	31.21 ± 9.24			
IL-6 sR (ng/mL) 5th	64	48.57 ± 10.07	52	49.68 ± 10.61	57	46.72 ± 10.2			
MPO (ng/mL) 1st	33	63.14 ± 28.91	51	77.26 ± 53.71	43	84.61 ± 51.55	-6.446 (4.372)	-6.877 (4.660)	-3.600 (2.305)
MPO (ng/mL) 2nd	74	90.94 ± 36.38	81	106.97 ± 56.87	66	117.94 ± 70.02			
MPO (ng/mL) 3rd	73	88.83 ± 47.01	74	105.16 ± 44.11	71	96.79 ± 43			
MPO (ng/mL) 4th	79	171.54 ± 80.19	68	166.01 ± 79.85	65	184.4 ± 94.49			
MPO (ng/mL) 5th	63	164.63 ± 96.94	51	186.14 ± 128.59	57	156.37 ± 102.1			
Arylesterase 1st	34	128.67 ± 29.24	51	128.32 ± 24.02	44	124.5 ± 25.05	-4.563 (1.741) ^★★^	-2869 (1.597)	-1.531 (0.789)
Arylesterase 2nd	74	87.33 ± 29.67	82	99.2 ± 30.37	67	96.48 ± 28.3			
Arylesterase 3rd	73	83.38 ± 13.19	74	80.23 ± 12.03	72	81.22 ± 12.16			
Arylesterase 5th	64	91.88 ± 15.35	52	95.51 ± 20.73	57	90.2 ± 16.5			

^a.^times of measurements (1st: at baseline; 2nd: at 6 months later; 3rd: at 18 months later; 4th: at 30 months later; 5th: at 42 months later)

★★ *p*-values interaction term <0.01 in the GEE model

Analytical Model (Dependent variable: IL-6) includes main effects of sex, and smoking.

Analytical Model (Dependent variable: IL-6 sR) includes main effects of age, sex, and smoking.

Analytical Model (Dependent variable: MPO) includes main effects of age, sex, smoking, drinking, arrhythmia, and hypertension.

Analytical Model (Dependent variable: Arylesterase) includes main effects of sex, and smoking.

**Table 7:** Generalized estimating equation analysis of ≥ 2 repeated measurements of cardiovascular disease markers over 4 years

	Control		Risk Level 1		Risk Level 2		GEE analysis coefficient B (SE)		
							Model 1		Model 2
Variables^a^	*N*	Mean ± SD	*N*	Mean ± SD	*N*	Mean ± SD	RL1*Time	RL2*Time	RL1*Time
paraoxonase (unit/ml) 1st	34	1121.64 ± 401.63	51	1196.94 ± 488.25	44	1205.68 ± 390.9	-10.619 (14.943)	-18.242 (12.892)	-9.259 (6.455)
paraoxonase (unit/ml) 2nd	74	900.4 ± 337.11	82	920.9 ± 358.07	67	936.96 ± 312.4			
paraoxonase (unit/ml) 3rd	73	869.72 ± 338.36	74	951.92 ± 298.78	72	915.69 ± 292.91			
paraoxonase (unit/ml) 4th	79	830.34 ± 325.89	68	920.14 ± 352.64	65	851.78 ± 314.39			
paraoxonase (unit/ml) 5th	64	779.12 ± 300.61	52	872.7 ± 341.33	57	847.03 ± 286.93			
SDNN (ms) 1st	34	39.09 ± 12.02	52	45.91 ± 17.25	45	43.57 ± 15.45	-1.541 (0.873)	-1.614 (0.887)	-0.817 (0.444)
SDNN (ms) 2nd	75	39.59 ± 16.06	81	43.57 ± 18.71	67	44.33 ± 17.26			
SDNN (ms) 3rd	73	43.64 ± 18.82	74	43.92 ± 17.18	71	44.2 ± 18.42			
SDNN (ms) 4th	80	39.25 ± 16.21	66	40.1 ± 14.12	65	39.35 ± 17.8			
SDNN (ms) 5th	62	39.6 ± 16.47	51	42.11 ± 16.31	57	37.65 ± 13.69			
RMSSD (ms) 1st	34	25.07 ± 11.06	52	32.92 ± 14.96	46	31.02 ± 15.77	-1.234 (0.716)	-1.203(0.763)	-0.596 (0.383)
RMSSD (ms) 2nd	73	27.4 ± 13.68	81	31.46 ± 18.8	66	30.56 ± 16.39			
RMSSD (ms) 3rd	72	29.34 ± 14.52	73	28.5 ± 15.21	70	28.84 ± 14.93			
RMSSD (ms) 4th	79	24.13 ± 11.42	67	27.09 ± 13.8	64	24.62 ± 12.9			
RMSSD (ms) 5th	65	23.6 ± 10.29	51	25.54 ± 12.35	57	24.88 ± 12.31			
VLF (ms2) 1st	34	595.94 ± 546.57	51	652.99 ± 516.35	44	568.88 ± 381.44	-35.240 (35.351)	-24.379 (33.075)	-12.450 (16.564)
VLF (ms2) 2nd	74	572.78 ± 560.28	79	655.26 ± 636.07	66	642.27 ± 536.73			
VLF (ms2) 3rd	71	573.79 ± 543.63	70	546.25 ± 448.1	65	561.91 ± 539.93			
VLF (ms2) 4th	78	510.31 ± 421.48	67	646.18 ± 601.7	61	520.77 ± 495.83			
VLF (ms2) 5th	59	642.16 ± 641.35	49	576.27 ± 522	55	532.74 ± 510.33			
LF (ms2) 1st	35	611.56 ± 480.14	52	694.45 ± 613.32	44	751.32 ± 739	-14.202 (30.830)	-53.531 (32.371)	-25.967 (16.115)
LF (ms2) 2nd	73	447.93 ± 386.22	79	575.7 ± 544.75	65	598.24 ± 536.82			
LF (ms2) 3rd	71	486.59 ± 495.96	73	554.67 ± 533.98	69	611.94 ± 594.38			
LF (ms2) 4th	78	424.3 ± 419.4	66	522.92 ± 448.81	62	508.42 ± 484.99			
LF (ms2) 5th	62	560.55 ± 540.79	50	657.62 ± 554.21	57	465.33 ± 445.06			
HF (ms2) 1st	34	235.81 ± 236.02	50	236.19 ± 171	45	296.39 ± 254.19	5.993 (10.816)	1.368 (12.301)	0.841 (6.119)
HF (ms2) 2nd	73	225.51 ± 189.97	77	259.41 ± 226.35	64	234.28 ± 141.02			
HF (ms2) 3rd	70	230.99 ± 202.36	70	261.96 ± 242.84	68	281.85 ± 236.15			
HF (ms2) 4th	76	185.35 ± 171.39	65	263.85 ± 246.1	63	253.78 ± 271.83			
HF (ms2) 5th	62	193.68 ± 165.35	50	254.4 ± 219.26	56	192.98 ± 167.61			
LF/HF ratio 1st	35	3.34 ± 1.96	51	3.04 ± 2.57	45	3.93 ± 3.62	0.026 (0.149)	-202 (0.162)	-0.105 (0.081)
LF/HF ratio 2nd	73	2.55 ± 2.09	78	2.69 ± 2.46	66	2.96 ± 2.53			
LF/HF ratio 3rd	71	2.55 ± 2.51	73	2.68 ± 2.56	72	3.05 ± 2.83			
LF/HF ratio 4th	77	2.76 ± 2.53	63	2.35 ± 1.79	62	2.75 ± 2.51			
LF/HF ratio 5th	61	3.09 ± 2.47	51	3.59 ± 3.21	56	3.23 ± 2.74			

^a^times of measurements (1st: at baseline; 2nd: at 6 months later; 3rd: at 18 months later; 4th: at 30 months later; 5th: at 42 months later)

Analytical Model (Dependent variable: paraoxonase) includes main effects of sex, and smoking.

Analytical Model (Dependent variable: SDNN) includes main effects of age, sex, smoking, chewing betel nut, and education.

Analytical Model (Dependent variable: RMSSD) includes main effects of age, sex, smoking, chewing betel nut, and education.

Analytical Model (Dependent variable: VLF) includes main effects of sex, and smoking.

Analytical Model (Dependent variable: LF) includes main effects of age, sex, smoking, hyperlipidemia, and education.

Analytical Model (Dependent variable: HF) includes main effects of age, sex, and smoking.

Analytical Model (Dependent variable: LF/HF ratio) includes main effects of sex, and smoking.

There were also no significant differences between the exposed and control workers for the changes of lung function test (Table 8 and 9), including forced vital capacity, forced expiratory volume at 1 second.0, forced expiratory flow at 25%, forced expiratory flow at 50%, and forced expiratory flow at 75%.

**Table 8:** Generalized estimating equation analysis of ≥ 2 repeated measurements of pulmonary function over 4 years

	Control		Risk Level 1		Risk Level 2		GEE analysis coefficient B(SE)		
							Model 1		Model 2
Variables^a^	*N*	Mean ± SD	*N*	Mean ± SD	*N*	Mean ± SD	RL1*Time	RL2*Time	RL1*Time
FVC (%) 1st	34	104.36 ± 10.86	53	106.17 ± 13.26	46	104.54 ± 13.2	1.164 (0.071)	0.468 (0.715)	0.269 (0.356)
FVC (%) 2nd	74	110.53 ± 20.59	81	109.51 ± 14.51	68	110.02 ± 15.39			
FVC (%) 3rd	73	106.18 ± 14.56	74	107.66 ± 12.05	72	104.48 ± 11.86			
FVC (%) 4th	77	108.14 ± 17.74	67	110.75 ± 14.62	64	107.49 ± 13.07			
FVC (%) 5th	65	106.06 ± 15.67	52	112.57 ± 15.93	57	107.94 ± 13.34			
FEV1.0/FVC (%) 1st	34	79.91 ± 7.24	53	82.23 ± 5.5	46	81.32 ± 6.43	-0.269 (0.328)	-0.084 (0.332)	-0.05 (1.673)
FEV1.0/FVC (%)2nd	74	81.43 ± 6.54	81	81.59 ± 4.92	68	81.47 ± 5.78			
FEV1.0/FVC (%) 3rd	73	80.49 ± 6.1	74	80.67 ± 5.45	72	82.02 ± 5.73			
FEV1.0/FVC (%) 4th	77	79.46 ± 6.93	67	79.47 ± 5.43	63	78.87 ± 6.07			
FEV1.0/FVC (%) 5th	65	80.34 ± 6.22	52	78.43 ± 5.56	57	79.57 ± 6.01			
MMF (%) 1st	34	83.29 ± 27.74	53	88.53 ± 21.09	46	84.69 ± 22.79	0.737 (1.084)	0.694 (1.082)	0.346 (0.542)
MMF (%) 2nd	74	91.41 ± 25.02	81	87.84 ± 20.43	68	91.6 ± 27.21			
MMF (%) 3rd	73	82.13 ± 22.35	74	83.45 ± 19.68	72	86.99 ± 21.13			
MMF (%) 4th	78	81.63 ± 20.93	67	81.69 ± 20.38	64	79.1 ± 22.4			
MMF (%) 5th	65	81.28 ± 21.85	52	79.7 ± 21.17	57	80.38 ± 22.13			
PEFR (%) 1st	34	99.03 ± 19.79	53	102.48 ± 13.82	46	99.93 ± 14.53	0.663 (0.889)	0.746 (0.962)	0.413 (0.480)
PEFR (%) 2nd	74	101.91 ± 17.9	81	102.86 ± 18.56	68	105.52 ± 20.51			
PEFR (%) 3rd	73	98.48 ± 15.69	74	105.89 ± 14.9	72	103.23 ± 16.68			
PEFR (%) 4th	78	94.96 ± 17.81	67	104.76 ± 12.95	64	99.08 ± 18.79			
PEFR (%) 5th	65	98.62 ± 15.35	52	104.23 ± 13.73	57	102 ± 20.13			

^a.^ times of measurements (1st: at baseline; 2nd: at 6 months later; 3rd: at 18 months later; 4th: at 30 months later; 5th: at 42 months later)

Analytical Model (Dependent variable: FVC) includes main effects of sex, smoking, allergic dermatitis, and education.

Analytical Model (Dependent variable: FEV1.0/FVC (%)) includes main effects of age, sex, smoking, asthma, and arrhythmia.

Analytical Model (Dependent variable: MMF (%)) includes main effects of sex, smoking, chronic bronchitis, and angina pectoris.

Analytical Model (Dependent variable: PEFR (%)) includes main effects of sex, smoking, chronic bronchitis, and angina pectoris.

**Table 9:** Generalized estimating equation analysis of ≥ 2 repeated measurements of pulmonary function over 4 years

	Control		Risk Level 1		Risk Level 2		GEE analysis coefficient B (SE)		
							Model 1		Model 2
Variables^a^	*N*	Mean ± SD	N	Mean ± SD	*N*	Mean ± SD	RL1*Time	RL2*Time	RL1*Time
FEF25(%) 1st	34	98.9 ± 24.62	53	103.5 ± 17.42	46	97.52 ± 17.93	1.269 (0.966)	1.337 (1.091)	0.717 (0.548)
FEF25(%) 2nd	74	103.91 ± 22.57	81	104.78 ± 18.88	68	105.08 ± 25.18			
FEF25(%) 3rd	73	96.11 ± 18.66	74	105.06 ± 18.88	72	101.56 ± 20.59			
FEF25(%) 4th	78	95.01 ± 19.87	67	104.29 ± 17.03	64	97.66 ± 20.74			
FEF25(%) 5th	65	97.02 ± 18.31	52	101.71 ± 17.88	57	98.54 ± 23			
FEF50(%) 1st	34	82.84 ± 25.1	53	87.64 ± 21.8	46	85.24 ± 21.66	1.002 (1.064)	0.549 (1.029)	0.305 (0.519)
FEF50(%) 2nd	74	90.64 ± 26.62	81	88.68 ± 20.19	68	91.92 ± 27.01			
FEF50(%) 3rd	73	83.09 ± 23.96	74	84.68 ± 19.41	72	87.51 ± 22.3			
FEF50(%) 4th	78	81.97 ± 21.32	67	84.03 ± 19.02	64	82.5 ± 22.02			
FEF50(%) 5th	65	80.15 ± 21.84	52	82.11 ± 21.41	57	81.84 ± 20.57			
FEF75(%) 1st	34	58.53 ± 26.86	53	64.07 ± 22.69	46	60.24 ± 25.06	-0.16 (1.230)	-0.04 (1.224)	-0.048 (0.613)
FEF75(%) 2nd	74	64.91 ± 22.94	81	61.12 ± 20.74	68	66.05 ± 25.75			
FEF75(%) 3rd	73	56.92 ± 19.78	74	57.15 ± 21.07	72	60.79 ± 17.83			
FEF75(%) 4th	77	56.91 ± 22.45	67	53.76 ± 17.94	64	51.62 ± 19.02			
FEF75(%) 5th	65	58.15 ± 24.74	50	51.74 ± 15.32	57	55.31 ± 23.12			

^a.^times of measurements (1st: at baseline; 2nd: at 6 months later; 3rd: at 18 months later; 4th: at 30 months later; 5th: at 42 months later)

Analytical Model (Dependent variable: FEF25 (%)) includes main effects of sex, smoking, chronic bronchitis, and angina pectoris.

Analytical Model (Dependent variable: FEF50 (%)) includes main effects of sex, smoking, and chronic bronchitis.

Analytical Model (Dependent variable: FEF75 (%)) includes main effects of age, sex, smoking, asthma, and arrhythmia.

## Discussion

Recently, several cases of illnesses were suspected of being caused by nanoparticles exposure and were reported in medical literatures. Two cases were reported in Germany and one in China, South Africa and Canada [47–52]. Although these cases have never been confirmed to be caused by inhalation of nanoparticles, there is increasing public, governmental, and scientific concerns on the potential adverse health effects of nanoparticle exposure. In order to avoid the selection bias of cross-sectional and short-term follow-up studies [53, 54], a long-term longitudinal study design with five repeated examinations in an interval of four years was designed to explore the potential adverse health effects of workers handling engineered nanoparticles. The researchers emphasize that this study was not to try to answer “What are the health effects of nanoparticles?” Instead, the study sought to answer “What are the potential adverse health effects among workers handling nanomaterials who are potentially exposed to nanoparticles?”

The findings of this four-year panel study showed that antioxidant enzymes, SOD, and GPx, were significantly associated with nanomaterial handling. However, cardiovascular dysfunction, lung damages, inflammation, oxidative damages, neurobehavioral and genotoxic markers were not found to be associated with nanomaterials handling in this panel study. Several possibilities can be used to explain the negative findings of health impact in this panel study. First, misclassification of exposure may lead to the underestimation of health effects. Since the researchers did not conduct environmental and personal sampling of nanoparticles, misclassification of exposure was possible. Second, negative confounders may underestimate the health effects and lead to no difference between exposed and control groups. Since the study evaluated and controlled confounders during data analysis for each effect marker, confounding bias was unlikely to be of concern in this study though some residual confounding could not be completely excluded. Third, loss to follow-up may lead to bias if loss to follow-up is associated with both exposure (risk levels) and disease (effect markers). Since loss to follow-up was not found to be associated with exposure (risk levels) in this study, loss to follow-up bias was not possible. Fourth, negligible exposure to engineered nanoparticles may lead to negative health impacts. Based on field studies conducted by Taiwan Institute of Occupational Safety and Health (IOSH) that measured the emission of nanoparticles in different operations or processes in nanotechnology industry (Table 10), nanoparticle emission was found to be negligible in enclosed operation of coating of nanomaterials, enclosed operation of mixing or grinding of nanopigments, and wet process in synthesis or centrifuge of nanomaterials. However, limited amount of nanoparticle emissions could be detected in spray drying of nanomaterials as well as dry process in polishing, milling and grinding. Table 1 indicates that most of the studied factories used liquid suspension and wet process. But there were still some factories using powder in operations, which may lead to the emission of small amounts of nanomaterials. Environmental monitoring in some of the studied plants showed that mass concentrations, surface area, and particles count of nanoparticles measured in post-operation were only slightly higher than those in pre-operation. The low concentrations of nanoparticles may induce an increase of early reaction of antioxidant enzymes, but the concentrations of nanoparticles were not high enough to induce oxidative damages and toxic effects of other organs. Therefore, nanomaterials handling in current negligible emission scenarios may not have health impact on workers in regards to cardiopulmonary injuries and oxidative damages, except for the increase of antioxidant enzymes that physiologically responds to negligible nanoparticle exposure. However, further study is needed to investigate whether exposure to higher concentration of nanoparticles or working longer in nanotechnology, other than operations or processes that were studied in this study, can cause health effects.

**Table 10:** Emission of nanomaterials in different operations or processes in nanotechnology industry

Operation	Emission of Nanomaterials
Coating of nanomaterials (Enclosed)	Negligible
Mixing/grinding of nanopigments (Enclosed)	Negligible
Synthesis of nanomaterials (Wet process)	Negligible
Centrifuge of nanomaterials (wet process)	Negligible
Spray drying of nanomaterials	Yes
Welding	Yes
Polishing	Yes
Milling	Yes
Grinding	Yes
Foundry Industry	Yes

Increase of antioxidant enzymes (SOD and GPX) was the only finding in this study. This finding was inconsistent with our previous cross-sectional study [20] and 6-month follow-up study [19]. In the cross-sectional study, we found that the antioxidant enzyme SOD in risk level 1 and risk level 2 workers was significantly (p < 0.05) lower than in control workers. A significantly decreasing gradient with risk levels was found for SOD (control > risk level 1 > risk level 2). Another antioxidant, glutathione peroxidase (GPX), was significantly lower only in risk level 1 workers than in the control workers. The cardiovascular markers, fibrinogen and ICAM, were significantly higher in risk level 2 workers than in controls, and a significant increasing gradient with risk levels was found for these two cardiovascular markers. Another cardiovascular marker, interleukin-6, was significantly increased among risk level 1 workers, but not risk level 2 workers [20]. While in the 6-month follow-up study, the researchers found that changes of the antioxidant enzymes (decrease of superoxide dismutase and glutathione peroxidase), cardiovascular markers (increase of vascular cell adhesion molecule, decrease of paraoxonase), the small airway damage marker (decrease of Clara cell protein 16), and lung function parameters (decrease of MMF, PEFR and FEF25%) were significantly associated with nanomaterial-handling during the 6-month follow-up period [19].

This discrepancy may be attributed to selection bias in cohort studies where the selection of study population into the cohort is somehow related to the probability of the outcome studied. An increased hazard in the time period after inclusion in the cohort may be caused by the study population with the outcome studied may be more prone to be recruited as members of the cohort [53, 54]. Selection bias of this type is likely to be more pronounced shortly after inclusion into the cohort. Cross-sectional study and short-term (e.g.6-month) follow-up study would have a high probability of suffering from this type of selection bias and result in positive health impacts. After some time period, the health problems leading to inclusion in the cohort could be resolved. Removal of observation time and events occurring shortly after inclusion in the cohort could reduce the impact of selection bias [53, 54]. In order to avoid such type of selection bias, this panel study used a long-term longitudinal study design with five repeated examinations in an interval of four years. The study concluded that nanomaterials handling in current negligible emission scenarios may not have health impact on the workers regarding cardiopulmonary injuries and oxidative damages, except for the increase of antioxidant enzymes physical response to the negligible emission of nanomaterials.

There are several limitations in this panel study. First, the small size of the study population limits its generalizability. The heterogeneity of the nanomaterials made it difficult to find a sufficiently large group of workers exposed to the same nanomaterial and to present potential health effects of any one nanomaterial. Second, misclassification of exposure by control banding was possible. Such random misclassification did not lead to systematic bias; random misclassification is biased toward the null value, which underestimates the true risk. Third, chronic effect markers should be used to evaluate the chronic, long-term health hazard of nanomaterials handling workers. Fourth, failure to set up the model for concurrent exposure to varied nanomaterials in order to understand the interaction among different nanomaterials. Finally, there is the risk to underestimate the effect of NPs exposure if workers with high exposures would not participate in the follow-up exams. This risk is low because we collected workers’ specimens during a series of annual health examinations, which provides the absence pattern of workers. However, we cannot fully exclude a healthy worker effect in which workers quit the job because of health issues before they started to participate in the study.

In order to prevent the hazards of handling nanomaterials, the introduction of strict preventive measures, such as local ventilation and personal protective equipment, are still the only way to prevent the risk of occupational disease in workers handling nanomaterials. Although negligible nanoparticle exposure was detected in the enclosed operation and the wet process of nanomaterials handling, limited amount of nanoparticles can still be detected in spray drying of nanomaterials as well as dry process in polishing, milling and grinding. Respiratory protection in addition to local ventilation should be implemented in nanotechnology in order to reduce nanoparticles exposure and to prevent nanoparticles-induced illnesses.

## Conclusions

An increasing gradient with risk levels of superoxide dismutase (SOD) and increase of glutathione peroxidase in RL 1 were found for nanomaterials handling workers in comparison to the controls. Industrial hygiene surveys and environmental emission surveys indicated that exposure concentrations of nanoparticles was dramatically reduced due to wet processes and liquid suspensions used in the workplace. Although nanomaterial handling in current low emission scenarios may not have 4-year health impact on the workers, long-term health effects of more than 5 years for nanomaterial used still need research.

## Data Availability

The datasets generated during and/or analysed during the current study are available from the corresponding author on reasonable request.

## Abbreviation

CC16: Clara cell protein
NO: Nitric oxide
NF-*kB*: Nuclear factor-kappa*B*
8-OHdG: 8-hydroxydeoxyguanosine
SOD: Superoxide dismutase
GPx: Glutathione peroxidase
VCAM: Vascular cell adhesion molecule
ICAM: Intercellular adhesion molecule
hsCRP: High sensitive C-reactive protein
MPO: Myeloperoxidase
IL-6: Interleukin-6
IL-6sR: Interleukin-6 soluble receptor
HRV: Heart rate variability
SDNN: Standard deviation of all normal to normal R-R intervals
RMSSD: The root mean square of successive differences between adjacent normal cycles
VLF: Very low frequency
LF: Low frequency
HF: High frequency
LF/HF: Low frequency/high frequency ratio
FVC: Forced vital capacity
FEV1: Forced expiratory volume at 1 second
MMF: Maximal mid-expiratory flow
PEFR: Peak expiratory flow rate
FEF25%: Forced expiratory flow at 25%
FEF50%: Forced expiratory flow at 50%
FEF75%: Forced expiratory flow at 75%
